# Immunotherapy: a promising approach for glioma treatment

**DOI:** 10.3389/fimmu.2023.1255611

**Published:** 2023-09-07

**Authors:** Feroza Yasinjan, Yang Xing, Huayue Geng, Rui Guo, Lei Yang, Ziling Liu, Hong Wang

**Affiliations:** ^1^ Cancer Center, The First Hospital of Jilin University, Changchun, China; ^2^ Department of Neurosurgery, The First Hospital of Jilin University, Changchun, China; ^3^ Clinical Laboratory, The First Hospital of Jilin University, Jilin University, Changchun, China

**Keywords:** immunotherapy, glioma, ICB, car-t, oncolytic viruses, vaccine, tumor microenvironment

## Abstract

Gliomas are the most prevalent primary malignant brain tumors worldwide, with glioblastoma (GBM) being the most common and aggressive type. Despite two decades of relentless pursuit in exploring novel therapeutic approaches for GBM, there is limited progress in improving patients’ survival outcomes. Numerous obstacles impede the effective treatment of GBM, including the immunosuppressive tumor microenvironment (TME), the blood-brain barrier, and extensive heterogeneity. Despite these challenges, immunotherapies are emerging as a promising avenue that may offer new hope for the treatment of gliomas. There are four main types of immunotherapies for gliomas, immune checkpoint blockades, chimeric antigen receptor T-cell therapies, vaccines, and oncolytic viruses. In addition, gene therapy, bispecific antibody therapy, and combine therapy are also briefly introduced in this review. The significant role of TME in the process of immunotherapies has been emphasized in many studies. Although immunotherapy is a promising treatment for gliomas, enormous effort is required to overcome the existing barriers to its success. Owing to the rapid development and increasing attention paid to immunotherapies for gliomas, this article aims to review the recent advances in immunotherapies for gliomas.

## 1 Introduction

Gliomas are the most prevalent primary malignant brain tumors worldwide. Gliomas often grow in the brain and come from glial tissue, yet they may form elsewhere in the central nervous system (CNS) (1–3). According to the up-to-date WHO classification of CNS tumors, diffuse gliomas in adults have been classified into: astrocytoma IDH-mutant (grade 2, 3, or 4), oligodendroglioma IDH-mutant and 1p/19q co-deleted (grade 2 or 3), and glioblastoma (GBM) IDH-wildtype (grade 4) (4). Moreover, patients diagnosed with GBM usually have a terrible prognosis, with a median overall survival (mOS) of less than two years, and a five-year survival rate of 10% (1–3).

The established treatment paradigm for GBM, known as the Stupp regimen, entails maximal surgical tumor resection followed by a combination of radiotherapy and chemotherapy (5). However, almost all patients show recurrence after receiving standard treatment. Thus, it is necessary to explore novel effective therapies for GBM. In recent years, immunotherapeutic strategies have revolutionized the treatment of various cancers, such as melanoma and lung cancer, and also bring new hope for GBM treatment (6–8).

Currently, more than 88 clinical trials on immunotherapies for GBM are being conducted worldwide (9). Moreover, the efficacy of several immunotherapeutic treatments, including the dendritic cell (DC) vaccine DCVax-L (10, 11) and oncolytic virus (OV) G47Δ (12), has been demonstrated in phase II and III clinical trials. And oncolytic virus G47Δ had also been conditionally approved in Japan for the treatment of malignant gliomas. These events have represented the potential power of immunotherapies in gliomas, and immunotherapies are worthy of our wait to change the bad prognosis of patients with GBM. However, several barriers, including blood-brain barrier (BBB), tumor microenvironment (TME), and substantial heterogeneity, broadly weaken and limit the efficacy of immunotherapies for gliomas. Therefore, novel practical therapeutic approaches are constantly being studied.

## 2 Immunotherapies in gliomas

Immunotherapies for cancer treatment refer to the engagement of patients’ immune systems to recognize and eliminate cancer. There are several kinds of immunotherapies used in cancer treatment, including immune checkpoint blockades (ICBs), adoptive cell therapies, therapeutic vaccines, OV therapies, etc. (13, 14). And different types of immunotherapies are applicable and suitable for different cancers. There are mainly four types of immunotherapies in treating gliomas: ICBs, Chimeric antigen receptor T (CAR-T) cell therapies, vaccines, and OVs (15, 16). ICB therapy can effectively block immune checkpoints such as PD-1/PD-L1 and thus inhibits the immunosuppressive effect. Peptide and dendritic vaccines are the immune targets of tumor-associated antigens (TAAs) and tumor-specific antigens (TSAs). CAR-T cell therapies targeting tumor surface molecules, such as EGFR variant III (EGFRvIII), IL13Rα2, and HER2, have also been explored in GBM treatment. OV therapy is an emerging treatment of GBM that has received widespread attention in recent years, with G47Δ being conditionally approved as a treatment option in Japan, opening the way for further development of immunotherapy (12). Next, we would like to analyze the recent advances in these major immunotherapeutic strategies.

### 2.1 ICBs

Immune checkpoints are surface molecules on immune cells that can regulate host immunity when they bind to the corresponding ligands or receptors on tumor cells or other cells (9). And tumor cells are proficient at employing this strategy to avoid the lethal effect exerted by immune cells (mainly T cells) (17). Immune checkpoint blockade (ICB) primarily refers to blocking the immunosuppressive immune checkpoints such as PD-1/PD-L1, and CTLA-4, thus inhibiting their corresponding immunosuppressive effects and playing an antitumor effect. And ICB has been proven feasible and effective in treating many cancers (18). However, there are no successful phase III clinical trials or marketing authorizations of ICBs in GBM treatment all over the world. Nevertheless, the exploration of this field is continuous and active, which can be indicated in **Table 1**. With a deeper understanding of the TME, some co-stimulatory checkpoints and their specific agonists have also been studied. Unlike other cancers, such as melanoma and lung cancer, the application of ICBs seems unfavorable in treating gliomas (19). Nevertheless, the combination therapy of ICB and other treatments, such as standard chemoradiotherapy, targeted therapies, or other different kinds of immunotherapies, may find a way to success (9).

**Table 1:** Recent phase II/III clinical trials of ICBs for GBM treatment.

Target	Identifier	Title	Phase	Treatment approach	Patient	Status
PD-1	NCT02017717 (CheckMate 143)	A randomized phase 3 open label study of nivolumab versus bevacizumab and multiple phase 1 safety cohorts of nivolumab or nivolumab in combination with ipilimumab across different lines of glioblastoma	3	Nivolumab Bevacizumab Ipilimumab	rGBM	Completed
	NCT02617589 (Checkmate 498)	A randomized phase 3 open label study of nivolumab vs. temozolomide each in combination with radiation therapy in newly diagnosed adult subjects with unmethylated MGMT (tumor o-6-methylguanine DNA methyltransferase) glioblastoma	3	Nivolumab Temozolomide Radiotherapy	GBM (Unmethylated MGMT)	Completed
	NCT02667587 (Checkmate 548)	A randomized phase 3 single-blind study of temozolomide, plus radiation therapy combined with nivolumab or placebo in newly diagnosed adult subjects with MGMT-methylated glioblastoma	3	Nivolumab Temozolomide Radiotherapy	nGBM	Active
	NCT02798406	Combination adenovirus + pembrolizumab to trigger immune virus effects	2	Pembrolizumab Adenovirus	rGBM	Completed
	NCT02550249	Phase 2 study of neoadjuvant nivolumab in patients with glioblastoma multiforme	2	Nivolumab	nGBM and rGBM	Completed
	NCT02336165	Phase 2 study to evaluate the clinical efficacy and safety of durvalumab (MEDI4736) in patients with glioblastoma (GBM)	2	Durvalumab	nGBM and rGBM	Completed
	NCT02337491	Phase 2 study of pembrolizumab (MK-3475) with and without bevacizumab for recurrent glioblastoma	2	Pembrolizumab Bevacizumab	rGBM	Completed
	NCT03018288	Radiation Therapy Plus Temozolomide and Pembrolizumab With and Without HSPPC-96 in Newly Diagnosed Glioblastoma (GBM)	2	Pembrolizumab Temozolomide Radiotherapy HSPPC-96	nGBM	Completed
	NCT03405792	Study Testing The Safety and Efficacy of Adjuvant Temozolomide Plus TTFields (Optune^®^) Plus Pembrolizumab in Patients With Newly Diagnosed Glioblastoma	2	Temozolomide TTFields Pembrolizumab	nGBM	Active
	NCT02337686	Pharmacodynamic study of pembrolizumab in patients with recurrent glioblastoma	2	Pembrolizumab	rGBM	Active
	NCT03452579	A randomized phase 2 open label study of nivolumab plus standard dose bevacizumab versus nivolumab plus low dose bevacizumab in recurrent glioblastoma (GBM)	2	Nivolumab Bevacizumab	rGBM	Active
	NCT03743662	Nivolumab With Radiation Therapy and Bevacizumab for Recurrent MGMT Methylated Glioblastoma	2	Nivolumab Bevacizumab Radiotherapy	rGBM (Methylated MGMT)	Active
	NCT03661723	Pembrolizumab and Reirradiation in Bevacizumab Naïve and Bevacizumab Resistant Recurrent Glioblastoma	2	Pembrolizumab Bevacizumab Radiotherapy	rGBM	Active
	NCT03665545	Pembrolizumab in Association With the IMA950/​Poly-ICLC for Relapsing Glioblastoma	1/2	Pembrolizumab IMA950/Poly-ICLC	rGBM	Active
	NCT04479241	LUMINOS-101: Lerapolturev (PVSRIPO) and Pembrolizumab in Patients With Recurrent Glioblastoma	2	Pembrolizumab Lerapolturev	rGBM	Active
	NCT04195139	Nivolumab and Temozolomide Versus Temozolomide Alone in Newly Diagnosed Elderly Patients With GBM (NUTMEG)	2	Nivolumab Temozolomide	nGBM (Elderly)	Active
	NCT04013672	Study of Pembrolizumab Plus SurVaxM for Glioblastoma at First Recurrence	2	Pembrolizumab SurVaxM	rGBM (at first recurrence)	Active
	NCT03890952	Translational Study of Nivolumab in Combination With Bevacizumab for Recurrent Glioblastoma	2	Nivolumab Bevacizumab	rGBM	Active
	NCT03899857	Pembrolizumab for Newly Diagnosed Glioblastoma (PERGOLA)	2	Pembrolizumab	nGBM	Active
	NCT03491683	INO-5401 and INO-9012 Delivered by Electroporation (EP) in Combination With Cemiplimab (REGN2810) in Newly-Diagnosed Glioblastoma (GBM)	1/2	Cemiplimab INO-5401 INO-9012	nGBM	Active
PD-L1	NCT03174197	Atezolizumab in Combination With Temozolomide and Radiation Therapy in Treating Patients With Newly Diagnosed Glioblastoma	1/2	Atezolizumab Temozolomide Radiotherapy	nGBM	Active
	NCT03750071	VXM01 Plus Avelumab Combination Study in Progressive Glioblastoma	1/2	Avelumab VXM01	Progressive GBM	Active
CTLA-4	NCT04817254	Phase II Trial Evaluating the Association of Peripheral Blood Immunologic Response to Therapeutic Response to Adjuvant Treatment With Immune Checkpoint Inhibition (ICI) in Patients With Newly Diagnosed Glioblastoma or Gliosarcoma	2	Ipilimumab Nivolumab TMZ	nGBM or Gliosarcoma	Recruiting
	NCT02794883	A phase 2, open label, clinical trial of pre surgical and adjuvant treatment of recurrent malignant glioma with tremelimumab and durvalumab (MEDI4736) alone and in combination to determine immunologic changes from treatment	2	Tremelimumab Durvalumab	Recurrent malignant glioma	Completed
	NCT03367715	Nivolumab, Ipilimumab, and Short-course Radiotherapy in Adults With Newly Diagnosed, MGMT Unmethylated Glioblastoma	2	Ipilimumab Nivolumab Radiotherapy	nGBM (Unmethylated MGMT)	Completed
	NCT04396860	Testing the Use of the Immunotherapy Drugs Ipilimumab and Nivolumab Plus Radiation Therapy Compared to the Usual Treatment (Temozolomide and Radiation Therapy) for Newly Diagnosed MGMT Unmethylated Glioblastoma	2/3	Ipilimumab Nivolumab Radiotherapy	nGBM (Unmethylated MGMT)	Active
IDO-1	NCT02052648	A phase 1/2 study of the combination of indoximod and temozolomide for adult patients with temozolomide-refractory primary malignant brain tumors	1/2	Indoximod Temozolomide	Recurrent malignant glioma	Completed

Data from ClinicalTrials.gov.

There are several completed clinical trials associated with ICBs for GBM treatment. All three clinical trials used anti-PD-1 monoclonal antibodies. PD-1 is a co-repressor molecule of the CD2 family and is expressed on the activated immune cells constitutively (20). After PD-1 binding to its ligands (mainly PD-L1) on tumor cells or antigen-presenting cells (APCs), T cells’ impotence, failure, or even apoptosis can be induced (21). In addition, PD-1 can also stimulate the proliferation of regulatory T cells (Tregs) and reduce the immune responses of natural killer cells and B cells (22).

Pembrolizumab (KEYTRUDA) is a commonly used monoclonal anti-PD-1 antibody. In 2019, satisfactory results of a randomized, multi-institutional trial of neoadjuvant pembrolizumab in patients with recurrent, surgically resectable GBM were published (23). Compared with patients receiving adjuvant, post-surgical administration of pembrolizumab alone, patients receiving neoadjuvant pembrolizumab and sustained adjuvant therapy after surgery had substantially improved OS and progression-free survival (PFS). The median OS of patients in the adjuvant-only group and the neoadjuvant group were 228 days (7.5 months) and 417 days (13.7 months), respectively, with a hazard ratio (neoadjuvant/adjuvant) of 0.39 (*P* = 0.04). And the median PFS of patients in the adjuvant-only group and the neoadjuvant group were 72.5 days (2.4 months) and 99.5 days (3.3 months), respectively, with a hazard ratio (neoadjuvant/adjuvant) of 0.43 (*P* = 0.03). The frequency of the focal upregulation of PD-L1 in the TME, the downregulation of PD-1 on T cells in the peripheral blood, and the intensive clonal expansion of T cells was higher in the neoadjuvant group compared with the adjuvant-only group. And the functional activation of tumor-infiltrating lymphocytes (TILs) was induced by neoadjuvant PD-1 blockade, which produced an interferon response within the TME (23).

The other two clinical trials applied another anti-PD-1 antibody, nivolumab (OPDIVO). Nivolumab is one of the most studied anti-PD-1 antibodies in treating gliomas. CheckMate 143 was the first phase III randomized clinical trial to test the efficacy of nivolumab in patients with recurrent GBM (rGBM) (24). But this trial’s primary endpoint (median OS, mOS) was not reached. Compared with the control group (bevacizumab) (10.0 months), the mOS was similar in the nivolumab group (9.8 months) (*P* = 0.76). And the PFS and the ORR (overall response rate) of the bevacizumab group were better than those of the nivolumab group. Besides, the post-subgroup analysis indicated that the potential benefits from nivolumab are more probable to be obtained in patients with methylated MGMT promoters (24). NCT02550249 was another crucial phase II clinical trial of nivolumab for GBM treatment. In this single-arm phase II clinical trial, twenty-seven patients with relapsed GBM and three patients with primary GBM were included to explore the feasibility, safety, and antitumor effects of neoadjuvant nivolumab in patients with resectable GBM (25). The neoadjuvant nivolumab was found to increase the expression of chemokines, infiltration of immune cells, and clonal diversity of T cell receptors in the TME (25).

There were also two completed randomized phase III clinical trials to test the efficacy of nivolumab in patients with newly diagnosed GBM (nGBM) (26, 27). The Checkmate 498 trial aimed to test the effectiveness of nivolumab plus radiotherapy, compared with TMZ plus radiotherapy, in patients with nGBM characterized as unmethylated MGMT promoters. However, its recently published results did not reach the primary endpoint (mOS). The mOSs of the nivolumab plus radiotherapy group and TMZ plus radiotherapy were 13.4 months and 14.9 months, respectively (27). The CheckMate 548 trial was a similar phase III study to evaluate the effectiveness of nivolumab plus the Stupp regime (radiotherapy plus TMZ), compared with the Stupp regime plus placebo, in patients with nGBM with methylated MGMT promoter (26). However, the results also showed a failure in adding nivolumab to the Stupp regime could not improve the OS and PFS of the patients. The mPFSs of nivolumab plus the Stupp regime group and the Stupp regime plus placebo group were 10.6 months and 10.3 months, respectively. And the mOSs were 28.9 months and 32.1 months, respectively.

In addition, some other immune checkpoints in gliomas, such as CTLA-4 (28–30), CD47 (31–33), CD73 (34, 35), TIGIT (36), and CD137 (37), were also studied in either clinical trials and preclinical research. It is worth noting that TILs in GBM are proven to highly express a number of co-inhibitory immune checkpoints, such as TIM-3, PD-1, and LAG-3, as a result of severe exhaustion of T cells (38). And this supports the necessity of exploring other feasible immune checkpoints and combined therapies to increase the effectiveness of the ICB (26, 39).

### 2.2 Vaccine therapy

Vaccines have a long history of use in cancer treatment (40, 41). Several types of vaccines are used in cancer treatment, and peptide and dendritic cell (DC) vaccines are the main strategies for glioma treatment (42–44). In addition, the TAA and TSA can be used as the immune targets of vaccines to stimulate adaptive immunity (45). **Table 2** lists the recent phase II/III clinical trials of vaccine therapy for GBM treatment from ClinicalTrials.gov.

**Table 2:** Recent phase II/III clinical trials of vaccine therapy for GBM treatment.

Vaccine type	Identifier	Title	Phase	Treatment approach	Patient type	Status
DC vaccine	NCT00045968	A phase 3 clinical trial evaluating DCVax-L, autologous dendritic cells pulsed with tumor lysate antigen for the treatment of glioblastoma multiforme	3	DCVax-L	nGBM	Active
	NCT03548571	Open label randomized phase 2/3 trial of dendritic cell immunotherapy against cancer stem cells in glioblastoma patients receiving standard therapy (DEN-STEM)	2/3	DEN-STEM	GBM (IDH wild-type, Methylated MGMT)	Active
	NCT00639639	Anti-tumor immunotherapy targeted against cytomegalovirus in patients with newly diagnosed glioblastoma multiforme during recovery from therapeutic temozolomide-induced lymphopenia	1/2	CMV-DC	nGBM	Completed
	NCT03688178	DC Migration Study to Evaluate TReg Depletion In GBM Patients With and Without Varlilumab	2	DERIVe	nGBM	Active
	NCT01204684	A Phase II Clinical Trial Evaluating Autologous Dendritic Cells Pulsed With Tumor Lysate Antigen +/- Toll-like Receptor Agonists for the Treatment of Malignant Glioma	2	DC vaccine Resiquimod polyICLC	Malignant glioma	Active
	NCT02465268	A Phase II Randomized, Blinded, and Placebo-controlled Trial of CMV RNA-Pulsed Dendritic Cells With Tetanus-Diphtheria Toxoid Vaccine in Patients With Newly-Diagnosed Glioblastoma	2	pp65 DC vaccine	nGBM	Active
	NCT00846456	Phase I/II Trial of Vaccine Therapy With Tumor Stem Cell Derived mRNA- Transfected Dendritic Cells in Patients Receiving Standard Therapy for Glioblastoma	1/2	Tumor stem cell derived mRNA- transfected DC vaccine Stupp regimen	GBM	Completed
	NCT00323115	A Phase II Feasibility Study of Adjuvant Intra-Nodal Autologous Dendritic Cell Vaccination for Newly Diagnosed Glioblastoma Multiforme	2	DC vaccine	nGBM	Completed
	NCT01006044	Prospective, Phase II Clinical Trial to Evaluate Efficacy and Safety of Autologous Dendritic Cell Vaccination in Glioblastoma Multiforme Patients After Complete Surgical Resection With Fluorescence Microscope	2	DC vaccine	GBM	Completed
	NCT01213407	First Line Standard Therapy of Glioblastoma Multiforme With or Without add-on Treatment With Trivax, an Anti-tumour Immune Therapy Based on Tumour-lysate Charged Dendritic Cells	2	Trivax	GBM	Completed
Peptide vaccine	NCT00458601	A phase 2 study of CDX-110 with radiation and temozolomide in patients with newly diagnosed GBM	2	Rindopepimut (CDX 110) TMZ Radiotherapy	nGBM	Completed
	NCT01480479	An international randomized double, blind, controlled study of rindopepimut/GM-CSF with adjuvant temozolomide in patients with newly diagnosed, surgically resected, EGFRvIII-positive glioblastoma	3	Rindopepimut (CDX 110) TMZ	nGBM; surgically resectedGBM; EGFRvIII-positive GBM	Completed
	NCT00905060	PHASE 2, Multi-center, Single Arm Investigation of HSPPC-96 Vaccine With Temozolomide in Patients With Newly Diagnosed Glioblastoma Multiforme	2	HSPPC-96 Temozolomide	nGBM	Completed
	NCT04280848	Anticancer Therapeutic Vaccination Using Telomerase-derived Universal Cancer Peptides in Glioblastoma	2	UCPVax	GBM	Active
	NCT02455557	A Phase II Study of the Safety and Efficacy of SVN53-67/M57-KLH (SurVaxM) in Survivin-Positive Newly Diagnosed Glioblastoma	2	SurVaxM Temozolomide	nGBM	Active
	NCT00643097	A Complementary Trial of an Immunotherapy Vaccine Against Tumor-Specific EGFRvIII	2	PEP-3-KLH conjugate vaccine	nGBM	Completed
	NCT01920191	Phase I/II Study of Intradermal IMA950 Peptide-based Vaccine Adjuvanted With Intra Muscular Poly-ICLC in Combination With Temozolomide in Newly Diagnosed HLA-A2 Glioblastoma Patients	1/2	IMA950 Poly-ICLC	GBM	Completed
	NCT00293423	Phase I/II Trial of Heat Shock Protein Peptide Complex-96 (HSPPC-96) Vaccine for Patients With Recurrent High Grade Glioma	1/2	GP96	Recurrent or progressive glioma	Completed
	NCT04116658	A Multicenter, Open-Label, First-in-Human, Phase 1b/2a Trial of EO2401, a Novel Multipeptide Therapeutic Vaccine, With and Without Check Point Inhibitor, Following Standard Treatment in Patients With Progressive Glioblastoma	1/2	EO2401	Recurrent or progressive glioma	Active
	NCT03665545	Pembrolizumab in Association With the Multipeptide Vaccine IMA950 Adjuvanted With Poly-ICLC for Relapsing Glioblastoma: a Randomized Phase I/II Trial	1/2	IMA950/Poly-ICLC Pembrolizumab	rGBM	Active

Data from ClinicalTrials.gov.

Peptide vaccines comprise 8-25 amino acids and have epitopes acting as antigenic targets (43). Moreover, they are usually conjugated to a carrier protein to increase immunogenicity. These vaccines with simple structures are relatively easy to manufacture and store and have relatively lower variability when compared to other vaccines (43, 46). The frequently mutated or highly expressed proteins or antigens in GBM mainly include EGFR, EGFRvIII, NF1, TERT, PDGFRA, PTEN, RB1, IDH1, TP53, PIK3R1, and PIK3CA, some of which are regarded as ideal vaccine targets (43, 47). EGFRvIII is a deletion mutation of EGFR, which can be detected in approximately 20% of GBM (48, 49). And EGFRvIII is proven to enhance tumor growth and chemotherapy resistance. Currently, EGFRvIII is one of the most studied TSAs and an important target in vaccines for GBM treatment (50). There was an important phase III clinical trial that used the peptide vaccine rindopepimut (51). However, the results of this phase III trial of rindopepimut in combination with TMZ in patients with EGFRvIII-positive nGBM did not show a not disappoint. Compared with the TMZ group, the rindopepimut plus TMZ group did not increase the mOS. The mOS of TMZ and rindopepimut plus TMZ groups were 20.1 months and 20.0 months, respectively (*P*=0.93). Besides, the result also indicated the necessity of multi-peptide vaccines against several targets to overcome the antigenic heterogeneity in GBM (43, 51).

DC vaccine is another studied type of vaccine for glioma treatment (52). Dendritic cells’ function as antigen-presenting cells serves as the foundation for the main mechanism of DC vaccines (53). When immune cells, especially T cells, are activated by DCs, they can cross BBB and enter the brain tumor site to play the antitumor effects (53, 54). And DCs are believed to trigger both innate and adaptive immune responses to facilitate the transformation of immunologically cold gliomas into immunologically hot gliomas (44, 53, 55). Currently, about half of the current phase II and III trials involving vaccines are cell-based strategies, especially DC vaccines (56).

In 2023, a phase III trial of an autologous tumor lysate-loaded DC vaccine (DCVax-L) plus TMZ in patients with nGBM and rGBM reported encouraging findings (10, 11). This study’s primary and secondary endpoints were the mOSs in patients with nGBM and rGBM, respectively (10). In the nGBM part, the mOSs in the DCVax-L plus TMZ group and the TMZ control group were 19.3 and 16.5 months, respectively (*P* = 0.002). And the survival rates of two and five years in DCVax-L plus TMZ group and TMZ control group were 15.7% vs 9.9% and 13.0% vs 5.7%, respectively (10). In the rGBM part, the mOSs in DCVax-L plus TMZ group and TMZ control group were 13.2 and 7.8 months, respectively (*P* < 0.001). And the survival rates of two years and 30 months of DCVax-L plus TMZ group and TMZ control group were 15.7% vs. 9.9% and 13.0% vs. 5.7%, respectively (10). Besides, DCVax-L-treated patients with nGBM with methylated MGMT promoter survived longer (21.3 months) than those in the external control group (*P* = 0.03) (10). Obviously, this phase III trial advances the pace of vaccine therapy for gliomas. However, the individual patient-level data of the external control populations are not accessible. And a more credible and reasonable investigation of DCVax-L in GBM treatment is needed.

### 2.3 CAR-T cell therapy

CAR-T cell therapy is a typical type of adoptive T-cell therapies (57). This therapeutic approach involves collecting T cells from a patient’s peripheral blood, followed by their modification, amplification, and activation to express CAR molecules on the cell membranes. These genetically engineered T cells are then administered to the patient through injection, allowing them to target specific tumor cell antigens (57). The significance of CAR-T cell therapy in treating gliomas has been identified, although it has yet to exhibit big success (58). **Table 3** lists the recent phase II/III clinical trials of CAR-T therapy for GBM treatment from ClinicalTrials.gov.

**Table 3:** Recent clinical trials of CAR-T cell therapy for GBM treatment.

Identifier	Title	Phase	Treatment approach	Patient type	Status
NCT01454596	A phase 1/2 study of the safety and feasibility of administering T cells expressing Anti-EGFRvIII chimeric antigen receptor to patients with malignant gliomas expressing EGFRvIII	1/2	EGFRvIII-CARs	Malignant glioma (EGFRvIII-positive)	Completed
NCT02208362	Genetically modified T-cells in treating patients with recurrent or refractory malignant glioma	1	IL13Rα2-CARs	Recurrent or refractory malignant glioma	Active
NCT01082926	Phase 1 study of cellular immunotherapy for recurrent/refractory malignant glioma using intratumoral infusions of GRm13Z40-2, an allogenic CD8+ cytolitic T-cell line genetically modified to express the IL13-zetakine and HyTK and to be resistant to glucocorticoids in combination with interleukin-2	1	GRm13Z40-2 interleukin-2	Recurrent/refractory malignant glioma	Completed
NCT01109095	Administration of HER2 chimeric antigen receptor expressing CMV-specific cytotoxic T cells in patients with glioblastoma multiforme (HERT-GBM)	1	HER2-CARs	Recurrent or progressive GBM	Completed
NCT05063682	A Phase 1 Study to Evaluate EGFRvIII -Targeted Chimeric Antigen Receptor (CAR) T Cells for Adult Patients With Leptomeningeal Glioblastoma	1	EGFRvIII-CAR T	leptomeningeal GBM	Active
NCT03726515	Phase 1 Study of EGFRvIII-Directed CAR T Cells Combined With PD-1 Inhibition in Patients With Newly Diagnosed, MGMT-Unmethylated Glioblastoma	1	CART-EGFRvIII Pembrolizumab	nGBM (Unmethylated MGMT, EGFRvIII-positive)	Completed

Data from ClinicalTrials.gov.

The phase I clinical trials associated with CAR-T cell therapy in GBM treatment shows that scientists are also actively trying in this field. In these clinical trials, several commonly used targeted antigens, including EGFRvIII (59), IL13Rα2 (60), and HER2 (61), had also been indicated. NCT02208362 was a phase I clinical trial of IL13Rα2-targeted CAR-T cell therapy in GBM treatment (60). In this study, two intracranial approaches were applied to deliver CAR-T cells to a patient with rGBM, including infusions into the resected tumor cavity and the ventricular system. And it was found that intraventricular therapy can achieve the wide regression of central nervous system tumors. In comparison, the intracavitary treatment seemed only to control the local tumor recurrence. Moreover, the CAR-T cell therapy provided the patient with a response for 7.5 months. Overall, the result proved that CAR-T cell therapy can show the antitumor activity in GBM treatment, and IL13Rα2 is a valid immunotherapeutic target for CAR-T cell therapy (60). NCT02209376 was another phase I study of CAR-T cell therapy in patients with EGFRvIII-positive rGBM (59). This first-in-human experiment of EGFRvIII-targeting CAR-T cells by intravenous administration proved that CAR-T cells could transfer to the brain tumor site. It is worth noting that the EGFRvIII level on tumor cells was observed to be reduced or eliminated after the CAR-T cell administration. Moreover, the effect of CAR-T cells on the TME in GBM was also emphasized in this study. The immunosuppressive Tregs were the dominant T cell type for TILs in the TME. And many immunosuppressive molecules, including PD-L1, IL-10, and IDO1, were also observed to increase. Besides, the study also indicated the combination therapy of CAR-T cell therapy and the inhibition of immunosuppressive pathways (59). In another phase I study (NCT01109095), researchers investigated the use of HER2-targeting CAR-modified virus-specific T cells (HER2-CAR VSTs) in patients with rGBM (61). Participants in this trial were administered one or more autologous HER2-CAR VST injections across five different dosage levels. Of the 17 patients, eight exhibited clinical benefits, with one showing a partial response and seven maintaining stable disease. The median overall survival (mOS) was 11.1 months following HER2-CAR VSTs administration and 24.5 months from the time of diagnosis (61).

CAR-T cell therapy has made a step forward in both hematologic and solid tumors. However, its use for GBM treatment remains limited due to the BBB, antigen escape, tumor heterogeneity, and TME (62). Though the mentioned three phase I clinical trials indicated firm hopes for GBM treatment, more clinical trials with larger samples and more reliable examinations are needed.

### 2.4 OV therapy

Oncolytic virus (OV) therapy has emerged as a significant treatment strategy and has become a focus of research in the field of oncology (63). In 2021, the Japanese Ministry of Health, Labor, and Welfare (MHLW) granted conditional and time-limited approval for G47Δ to treat patients with malignant glioma in Japan (12). **Table 4** presents a summary of recent phase II/III clinical trials for OV therapy in the treatment of glioblastoma (GBM), as found on ClinicalTrials.gov.

**Table 4:** Recent phase II/III clinical trials of OV therapy for GBM treatment.

Identifier	Title	Phase	Treatment approach	Patient type	Status
NCT00028158	An Open-Label Phase Ib/II Study of the Safety, Tolerability and Efficacy of G207, a Genetically Engineered Herpes Simplex Type-1 Virus, Administered Intracerebrally to Patients With Recurrent Malignant Glioma	1/2	HSV G207	Recurrent malignant glioma	Completed
NCT01301430	Phase I/IIa Study of Intratumoral/Intracerebral or Intravenous/Intracerebral Administration of Parvovirus H-1 (ParvOryx) in Patients With Progressive Primary or Recurrent Glioblastoma Multiforme.	1/2	ParvOryx	Recurrent or progressive GBM	Completed
NCT01956734	Phase I Trial of Combination of DNX-2401 (Formerly Named Delta-24-RGD) Oncolytic Adenovirus With a Short Course of Temozolomide for Treatment of Glioblastoma at First Recurrent	1	DNX-2401 Temozolomide	First recurrent GBM	Completed
NCT02197169	A Phase 1b, Randomized, Multi-center, Open-label Study of a Conditionally Replicative Adenovirus (DNX-2401) and Interferon Gamma (IFN-γ) for Recurrent Glioblastoma or Gliosarcoma (TARGET-I)	1	DNX-2401 IFN-γ	rGBM or Gliosarcoma	Completed
NCT02062827	A Phase 1 Study of M032 (NSC 733972), a Genetically Engineered HSV-1 Expressing IL-12, in Patients With Recurrent/Progressive Glioblastoma Multiforme, Anaplastic Astrocytoma, or Gliosarcoma	1	M032	Recurrent/progressive GBM multiforme, anaplastic astrocytoma, or gliosarcoma	Active
NCT03072134	Neural Stem Cell Oncolytic Adenoviral Virotherapy of Newly Diagnosed Malignant Glioma	1	NSC-CRAd-S-p7 Adiation/chemotherapy	Newly diagnosed malignant glioma	Completed
NCT02798406	A Phase II, Multi-center, Open-label Study of a Conditionally Replicative Adenovirus (DNX-2401) With Pembrolizumab (KEYTRUDA^®^) for Recurrent Glioblastoma or Gliosarcoma (CAPTIVE/KEYNOTE-192)	2	DNX-2401 pembrolizumab	rGBM or Gliosarcoma	Completed
NCT02457845	Phase I Clinical Trial of HSV G207 Alone or With a Single Radiation Dose in Children With Recurrent Supratentorial Brain Tumors	1	HSV G207 Radiotherapy	Recurrent malignant glioma (Supratentorial)	Active
NCT00528684	A Phase I/II Clinical Trial to Evaluate Dose Limiting Toxicity and Efficacy of Intralesional Administration of REOLYSIN^®^ for the Treatment of Patients With Histologically Confirmed Recurrent Malignant Gliomas	1	REOLYSIN	Recurrent malignant glioma	Completed

Data from ClinicalTrials.gov.

OVs are genetically modified, weakly pathogenic viruses that enhance the antitumor effects without harming normal cells (63). OV therapy hasadvantage in this era of mature genetic engineering. There are three purposes for genetic modification of OVs: 1) delete virulence genes to improve the safety of OVs, 2) enhance the tumor cell tropism and targeting of OVs to tumor cells, and 3) modify OVs with different therapeutic genes to enhance anti-tumor effects (64). On the one hand, OVs can self-replicate in host cancer cells, leading to direct lysis of the cancer cells (63). On the other hand, OVs help activate innate and adaptive immune responses by the releases of damage-related molecular patterns (DAMPs), viral pathogen-associated molecular patterns (PAMPs), and TAAs, to improve the immunosuppressive TME (64–67). Besides, OVs can selectively target and inhibit glioma stem cells (GSCs), which is an essential factor for drug resistance, tumor blood vessel formation, and immunosuppressive glioma microenvironment (68–70).

Since gliomas develop predominantly in the brain and lack distant metastases, which allows for the viruses that need an active cell cycle for reproduction, gliomas are particularly well-suited for OV treatment (71). The viruses used for OV therapy in gliomas include Oncolytic H-1 Parvovirus (72), Oncolytic Reovirus (73, 74), Oncolytic measles virus (75), Newcastle disease virus (74), Oncolytic vaccinia virus (76), Poliovirus (77), Oncolytic adenovirus (78), Oncolytic zika virus (79), Oncolytic herpes simplex virus (64, 80). Moreover, of all the OVs, oHSV progresses furthest in the clinical practice, including G47Δ, G207, HSV1716, and rQNestin-34.5 (80).

UMIN000015995 was a phase II trial of G47Δ in Japan for patients with residual or recurrent GBM (12). The third-generation as well as the triple-mutated oncolytic herpes simplex virus type 1 (HSV-1) G47 was created by deleting the US11 promoter from its parental G207 and overlapping the US11 gene (81). The primary endpoint (1-year survival rate) was achieved ahead of schedule, which was 84.2%. Additionally, the secondary endpoints of OS and PFS were 20.2 months and 4.7 months, respectively, after the G47 initiation (12). Based on the exciting results, G47Δ obtained conditional and time-limited approval in Japan for malignant glioma patients (12).

As a promising cancer treatment approach, OV therapy has a significant effect on gliomas. Moreover, the successful application of G47Δ in Japan also indicates the huge potential of OV therapy. Nevertheless, further research and phase III trials with large samples are needed to verify the real efficacy of this novel treatment approach.

### 2.5 Other therapies

#### 2.5.1 Gene therapy

Gene therapy, a rapidly evolving field in oncology, encompasses the techniques used to introduce exogenous genes into targeted cells or tissues. These interventions are aimed at correcting or compensating for genetic defects and abnormalities for therapeutic purposes (82). Vectors in gene therapy can deliver entire genes, gene regulatory elements, or oligonucleotides and are categorized into viral and non-viral types (83). Viral vectors, including adenovirus vectors (AdV), adeno-associated viral vectors (AAV), retrovirus vectors (RV), and lentiviral vectors (LV), are non-toxic purified viruses designed to deliver genetic payloads without causing infections (84–88). Recently, non-viral vector-mediated gene therapy, such as nanoparticles with low toxicity and immunogenicity, has emerged as a promising approach in GBM treatment. These non-viral vectors can efficiently traverse the BBB and deliver gene drugs for glioma therapy (89, 90). The primary strategies in gene therapy are suicide gene therapy, tumor suppressor gene therapy, gene target therapy, and immunomodulatory gene therapy (91). Suicide gene therapy involves the delivery of genes coding specific enzymes that convert or activate non-toxic prodrugs into cytotoxic drugs at tumor sites, resulting in oncolytic action and tumor cell apoptosis (92). Tumor suppressor gene therapy focuses on restoring normal tumor suppressor gene function in tumor cells, thereby inhibiting tumor growth through genes like TP53, p16, and PTEN (93). Gene-targeted therapy binds specific tumor antigens and blocks carcinogenic pathways (91), while immunomodulatory gene therapy regulates the immunosuppressive tumor microenvironment (TME) and enhances immune cell effects. This therapeutic approach can be either categorized into gene therapy or immunotherapy. Moreover, with the development of both gene therapy and immunotherapy, the two approaches have become more closely linked. One good example is OV-based immunotherapy. Natural viruses can be genetically modified through genetic engineering into special OVs, which can specifically recognize, infect, and replicate in tumor cells, thereby destroying the tumor cells (71). Besides, tumor vaccines and CAR-T therapies can also be regarded as gene therapies in some situations.

#### 2.5.2 Bispecific antibody therapy

In recent years, bispecific antibodies (BsAbs) have emerged as a promising therapeutic approach in oncology, with notable applications in cancer treatment (94, 95). Unlike conventional antibodies, BsAbs possess two distinct antigen-binding sites, allowing them to function through various mechanisms, including immune cell activation, co-inhibitory receptor blockade, co-stimulatory molecule triggering, signaling pathway suppression, and collaborative targeting of cancer-related antigens (96). Several BsAbs, such as blinatumomab, mosunetuzumab, teclistamab, epcoritamab, and glofitamab, have gained approval for cancer treatment (95). However, the development of BsAbs for glioma therapy has been relatively slow, with most investigations confined to preclinical stages (97–101). One particular focus of this review is AG596, a bispecific T-cell engager (BiTE) that has entered phase I clinical trials, offering insight into the mechanism of BsAbs in oncological applications (102, 103). BiTEs represent a distinct class of BsAbs, comprised of two single-chain variable fragments (scFv) connected by a short peptide linker. Each scFv serves a specific function: one targets a tumor-associated antigen on tumor cells, while the other binds to CD3 expressed on T cells. The design of BiTEs in glioma treatment often involves EGFRvIII, similar to vaccine and CAR-T therapies (98–101). AG596, for instance, can simultaneously engage the tumor-specific antigen (EGFRvIII) on glioblastoma (GBM) cells and CD3 on T cells, thereby activating T cell proliferation and cytotoxic secretion. This process leads to T cell-mediated GBM cell destruction. Preclinical studies have demonstrated that AG596 effectively mediates the lysis of EGFRvIII-positive GBM cell lines, improving overall survival (OS) rates in mice bearing EGFRvIII-expressing GBM cells (100). Moreover, the selectivity of AMG 596 has been confirmed through testing. Its binding was specific to EGFRvIII-positive GBM cells, with no observed T cell activity in EGFRvIII-negative cells and no toxicity detected in normal tissues (EGFRvIII-negative) in cynomolgus monkeys (100). A phase I, first-in-human, sequential dose-escalation/expansion study of AMG 596 in patients with EGFRvIII-positive GBM or malignant glioma (either recurrent or newly diagnosed) (NCT03296696) provided preliminary evidence for its safety, tolerability, and anti-tumor activity in recurrent GBM (104). However, further research is required to fully understand and harness the potential of AMG 596 in GBM therapy. Other ongoing clinical trials of BiTEs for glioma treatment include NCT04903795 and NCT03344250 (phase I).

#### 2.5.3 Combined therapies

GBM is recognized as a highly malignant brain tumor, characterized by pronounced heterogeneity, an immunosuppressive TME, and a propensity for recurrence (105, 106). This complexity has necessitated the adoption of combined therapies, a strategy that neurosurgeons have embraced early on. The Stupp regimen, combining surgery with chemotherapy and radiotherapy, is a prototypical example (5). The emergence of novel treatments, including immunotherapy, targeted therapy, and tumor treatment fields, has given rise to innovative combinations, showing promise in both preclinical and clinical settings. Several tables from sections 2.1 to 2.4, along with most of our discussions, touch upon these combined therapies. This section would like to briefly introduce several prevalent combined therapeutic modes that incorporate immunotherapies in both clinical practice and trials.

Immunotherapy combined with chemotherapy (especially temozolomide (TMZ)). This approach is commonly used in clinical trials, and immunotherapy combined with current standard therapy (Stupp Regimen) can also be categorized into this category. Examples of clinical trials are Checkmate 498 and Checkmate 548 (using nivolumab, TMZ, and radiotherapy), NCT03018288 (Pembrolizumab, Temozolomide Radiotherapy, and HSPPC-96), NCT03548571, NCT00458601 (Rindopepimut, TMZ, and radiotherapy), NCT01480479 (Rindopepimut and TMZ), NCT02455557 (SurVaxM and TMZ), NCT01956734 (DNX-2401 and TMZ), etc.

Immunotherapy combined with targeted therapy (frequently paired with bevacizumab). This combined approach has also been explored in clinical trials such as CheckMate 143 (nivolumab and bevacizumab), NCT02337491 (Pembrolizumab and Bevacizumab), and NCT03452579 (Nivolumab and Bevacizumab), etc.

Combination of different types of immunotherapies or immunotherapeutic drugs (in the same type). Examples of combined immunotherapies in the same categories mainly include NCT02794883 (Tremelimumab and Durvalumab), NCT04396860 (Ipilimumab, Nivolumab, and Radiotherapy), etc. And examples of combined immunotherapies in different categories include NCT02798406 (Pembrolizumab and Adenovirus), NCT03750071 (Avelumab and VXM01), NCT01082926 (GRm13Z40-2 and IL-2), NCT03726515 (CART-EGFRvIII and Pembrolizumab), NCT02798406 (DNX-2401 and pembrolizumab), etc.

Given the current landscape of immunotherapies in gliomas, it is anticipated that more combined therapies yielding convincing results will emerge in the near future. These combined modalities hold the potential to address the multifaceted challenges posed by GBM, offering a more comprehensive approach to treatment.

## 3 TME in gliomas

As introduced above, immunotherapies have demonstrated promising outcomes in preclinical and clinical studies, especially the successful phase III trial of DCVax-L and the approval of G47Δ in Japan. However, there are also many obstructions on the way to success. The tumor microenvironment (TME) is regarded as one of the most critical factors in various treatments, including immunotherapy. On the one hand, the immunosuppressive TME in gliomas can result in drug resistance and tumor recurrence. On the other hand, understanding deeply and making great use of the TME can also promote the progress of immunotherapy in gliomas.

**Figure 1:**
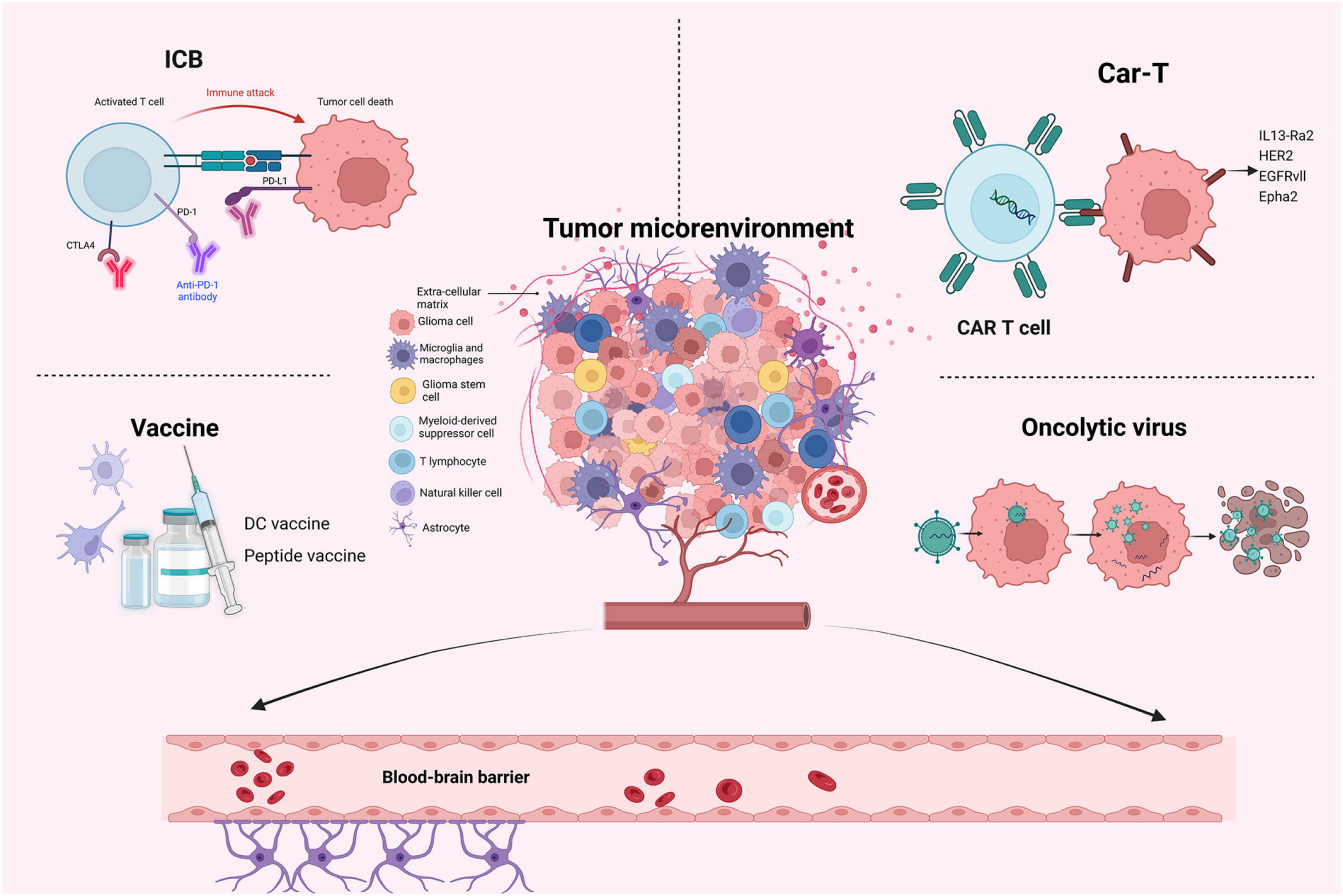
The illustration of four main immunotherapies and TME in gliomas.

The TME of gliomas is complex and heterogeneous, consisting of various components, including astrocytes, pericytes, endothelial cells, GSCs, blood vessels, glioma-associated stromal cells, immune cells including myeloid-derived suppressor cells (MDSCs), glioma-associated microglia/macrophages (GAMs), CD4^+^ T cells, Tregs and NK cells, and extracellular matrix (ECM) (107–109) (**Figure 1**). These elements may interact with one another to promote the spread and proliferation of glioma cells. It is widely acknowledged that TME is one of the main reasons for the unsatisfactory immunotherapeutic effect of gliomas. Additionally, more and more immunotherapy-related research pays much attention to TME in gliomas (23, 25, 59).

The powerful immunosuppressive effect in the TME of gliomas is caused by several mechanisms, including the abnormal function of cells such as the existence of immunosuppressive cells (M2 GAMs, Tregs, and MDSCs) and immunosuppressive cytokines (TGF-β, IL-10), low number of TILs, and the high expression of inhibitory immune checkpoint molecules such as PD-1, TIM-3, and LAG-3 (38, 109–112). The most abundant cells in GBM are GAMs, which are divided into the M1 phenotype (proinflammatory) and M2 phenotype (immunosuppressive) (107). These GAMs contribute to tumor heterogeneity and progression. On the one hand, M2 GAMs can produce a lot of IL-10 and TGF-β and low levels of IL-12, which can play an immunosuppressive role in the TME (109). GAMs can also promote the proliferation of GSCs, which is closely related to drug resistance (113).

Compared with other tumors, research on TILs in gliomas is relatively less (110). After T cells infiltrate the TME, dysfunction occurs through different mechanisms, including aging, tolerance, and incompetence (111). Additionally, glioma cells and certain immune cells emit a variety of immunosuppressive substances into the TME, including TGF-β and IL-10. Then these factors attract and stimulate immunosuppressive cells such as TAMs and Treg cells and inhibit the activation of APCs and effector immune cells (114, 115).

Glioma stem cells (GSCs), namely cancer stem cells in gliomas, also play a vital role in the TME. GSCs have the ability of self-renewal and multi-differentiation, which are regarded as the primary cause of tumor occurrence, development, drug resistance and recurrence, and heterogeneity (116–118). GSCs can regulate cell metabolism in the TME of gliomas to increase resistance to challenging conditions in addition to reprogramming associated cells to promote tissue remodeling (116–118). The intimate interaction between GSCs and glioma TME is a critical factor to the resistance of immunotherapy, and targeting both GSCs and TME can enhance the immunotherapeutic effect.

The BBB is also found closely connected with the immune response in gliomas. BBB is a semi-permeable physiological boundary formed by parenchymal capillary endothelial cells, capillary astrocytic endfeet, and capillary basal membrane pericytes (119, 120). The presence of BBB benefits the central nervous system’s special immune privilege, which is characterized by the lack of traditional lymphatic structures, a dearth of APC, low levels of MHC molecule expression, and the constitutive expression of immunosuppressive cytokines like IL10 and TGF-β (121–123). Moreover, BBB can also largely limit the delivery of most therapeutic drugs in treating gliomas (124). In addition to being a barrier to both immunity and drug delivery, several studies on tumor brain metastasis proved that the properties of BBB can be fundamentally regulated by some components of the TME (125).

## 4 Conclusion and prospect

This review analyzed the present landscape of immunotherapies for gliomas, focusing on four main therapeutic approaches: ICBs, CAR-T cell therapy, vaccine therapy, and OV therapy. Among these, the efficacy of the dendritic cell vaccine DCVax-L and OV G47Δ has been validated in phase II and III clinical trials, respectively. Notably, G47Δ received conditional and time-limited approval from the Japanese Ministry of Health, Labor, and Welfare (MHLW) in 2021 for the treatment of malignant glioma. This review also briefly introduces gene therapy and bispecific antibody therapy, both closely aligned with immunotherapy, as well as combined therapies incorporating immunotherapy. The critical role of the tumor microenvironment (TME) in the effectiveness of immunotherapies for gliomas has been emphasized in this review.

Immunotherapy, given its present standing and rapid evolution, is heralded as a promising avenue for glioma treatment. However, significant challenges must be overcome to realize its full potential. These include:

Understanding Molecular Biology: Investigations into the molecular biology of GBM, TME, and the BBB are vital for designing innovative immunotherapeutic drugs with potential antitumor effects.
Exploring TME-Immunotherapy Relationships: Continued and profound exploration of the relationship between TME and immunotherapy is necessary for the success of immunotherapies in gliomas.
Combination Therapies: Active and rational exploration of combined therapies is likely to become the focal point in this field, offering synergistic advantages.
Personalized Treatment: Tailoring treatments to individual patients with distinct molecular characteristics may be essential to optimize therapeutic outcomes.

In conclusion, while the rapid advancement of immunotherapy provides hope, the complexities of gliomas necessitate an equally nuanced approach to treatment. We ardently hope that immunotherapy, with continued research and development, will become a formidable tool in the fight against GBM in the foreseeable future.

## Author contributions

FY: Data curation, Formal Analysis, Funding acquisition, Investigation, Methodology, Writing – original draft, Writing – review & editing. YX: Funding acquisition, Writing – review & editing, Data curation, Formal Analysis, Investigation, Methodology, Writing – original draft. HG: Data curation, Investigation, Methodology, Writing – original draft. RG: Supervision, Writing – review & editing, Conceptualization, Supervision. LY: Supervision, Writing – review & editing, Conceptualization, Supervision. ZL: Supervision, Writing – review & editing, Conceptualization, Supervision. HW: Conceptualization, Funding acquisition, Resources, Supervision, Writing – review & editing.
